# Uncontrollable behavior or mental illness? Exploring constructions of bulimia using Q methodology

**DOI:** 10.1186/s40337-014-0022-2

**Published:** 2014-07-29

**Authors:** Kate Churruca, Janette Perz, Jane M Ussher

**Affiliations:** Centre for Health Research, University of Western Sydney, Locked Bag 1797, Penrith, South 2751 Australia

**Keywords:** Bulimia, Social constructionism, Q methodology, Help-seeking

## Abstract

**Background:**

In medical and psychological literature bulimia is commonly described as a mental illness. However, from a social constructionist perspective the meaning of bulimia will always be socially and historically situated and multiple. Thus, there is always the possibility for other understandings or *constructions* of bulimia to circulate in our culture, with each having distinct real-world implications for those engaging in bulimic behaviors; for instance, they might potentially influence likelihood of help-seeking and the success of treatment. This study used Q methodology to explore culturally-available constructions of bulimia nervosa.

**Methods:**

Seventy-seven adults with varying experience of eating disorders took part in this Q methodological study. Online, they were asked to rank-order 42 statements about bulimia, and then answer a series of questions about the task and their knowledge of bulimia. A by-person factor analysis was then conducted, with factors extracted using the centroid technique and a varimax rotation.

**Results:**

Six factors satisfied selection criteria and were subsequently interpreted. Factor A, “bulimia as uncontrolled behavior”, positions bulimia as a behavioral rather than psychological issue. Factor B, entitled “bulimia is a distressing mental illness”, reflects an understanding of bulimic behaviors as a dysfunctional coping mechanism, which is often found in psychological literature. Other perspectives position bulimia as about “self-medicating with food” (Factor C), “the pathological pursuit of thinness” (Factor D), “being the best at being thin” (Factor E), or as “extreme behavior vs. mentally ill” (Factor F). These constructions have distinct implications for the subjective experience and behavior of those engaged in bulimic behaviors, with some constructions possibly being more useful in terms of help-seeking (Factor B), while others position these individuals in ways that may be distressing, for instance as shallow (Factor D) or to blame (Factor E).

**Conclusions:**

This study has identified a range of distinct constructions of bulimia. These constructions are considered to have implications for the behaviors and experiences of those engaging in bulimic behaviors. As such, further research into constructions of bulimia may illuminate factors that influence help-seeking and the self-perceptions of such individuals.

## Background

Clinically diagnosable bulimia nervosa can be identified in approximately one in every hundred young women in Western societies [1,2]. There is evidence that the behaviors that characterize this condition, such as binge eating and purging, are becoming increasingly common, with a more than twofold rise in prevalence for both men and women between 1995 and 2005 [1]. Despite this general increase, bulimic behaviors still occur more commonly in women than in men [1,3,4]; as such, bulimia is viewed as predominately a woman’s problem [5]. However, most women exhibiting bulimic behaviors do not seek help or treatment [6–8]. This is a matter of serious concern, given the numerous associated health risks, such as cardiovascular and gastrointestinal problems [9,10], as well as psychological distress and reduced quality of life [11–13]. As such, there is a need for research that examines factors that may influence help-seeking for bulimic behaviors.

A variety of factors have been implicated in the development and maintenance of bulimia, which can be broadly characterised as biological [14], psychological [15,16] and sociocultural [5,17]. However, bulimia is a complex problem, and is not easily explained through one particular factor, or type of factor [18]. Instead, integrating factors together within a multifactorial bio-psycho-social model is considered necessary to more fully understand eating disorders [18–21]. For such an approach to be truly integrative, however, it should not only focus on the way social, psychological and biological factors intersect to determine eating disordered behaviors. Rather, as is recommended in the study of all categories of health and illness [22,23], it should also examine the way social meanings of eating disorders affect subjective experience. Research into bulimia has generally focused on the multifactorial aetiology of the behavior [24], rather than the social meaning [25]. This is typical for investigations into psychiatric diagnostic categories [26], as causal pathways and behaviours are more readily observed and quantified than meaning [22], and statistical analysis of such pathways is highly valued in psychological and medical research [27]. However, social constructionism provides a theoretical basis from which to explore the variety of social meanings of bulimia, and the implications that these different understandings have for those involved.

Proponents of social constructionist theory argue that meaning and knowledge are constructed in ongoing processes of social interaction [28]. As such, one’s perspective on a concept, such as bulimia, is always socially and historically situated, and open to being contested by competing understandings [28]. Bulimia might therefore be understood in terms of a number of alternate *constructions*: these are coherent accounts, representations, or ways of thinking about a topic, that circulate widely in our culture [29]. This includes biomedical, psychological and many social theories of eating disorders, that despite their differences, reflect a dominant construction that circulates in research, popular culture, and self-help resources, in which bulimia is positioned as an individual pathology and categorically distinct from normal eating behaviors [30–32].

These different constructions have different real-world implications for the subjectivity of those individuals engaging in bulimic behaviors; social constructionism suggests that they frame feelings, experiences, sense of self and perceptions of one’s own behavior, making certain actions possible, while simultaneously closing down others [23,29,33]. For instance, the construction of bulimia as a mental illness, a common conceptualisation in the research literature [18,34], positions individuals with a bulimic diagnosis as not responsible for the abnormal and distressing condition they experience, and opens up the possibility for particular forms of treatment. On the other hand, as has been suggested for women diagnosed with depression [35,36], this construction might minimize the potential for personal agency and recovery without such treatment. This understanding may also conflict with other constructions of bulimia, as suggested in a study of community attitudes to mental illness in the UK, where over one-third of participants considered individuals with eating disorders responsible for their condition and that they should “pull themselves together” [37]. A further contrasting understanding of bulimia is found in some feminist theorising, where behaviors categorised as eating disorders are viewed as part of a continuum of eating behaviors, with psychiatric diagnosis of particular behaviors as ‘disordered’ deemed to reflect a broader cultural objectification and pathologization of women’s bodies, linked to cultural constructions of normative femininities [32].

Research driven by social constructionist theory has previously been used to explore some aspects of bulimia. For instance, social constructionism has highlighted the negative relationship between cultural constructions of bulimia and anorexia, with the former having connotations of being out-of-control and disgusting, whereas the latter is partially glamorised and admired [38–40]. Evidence of this binary has been found in the talk of both health professionals and individuals engaged in bulimic behaviors, which may contribute to distress experienced by the latter and potentially intensify their dysfunctional eating behaviors [38]. Finally, the identity of “the bulimic” may efface other aspects of self; as such, this construction may limit willingness for treatment, with recovery becoming akin to “self-annihilation” [25].

If constructions of eating disorders impact upon the experiences of those engaged in bulimia, with the potential to impact upon behaviours such as help-seeking, it is important to identify and explicate dominant constructions which operate in a particular historical and cultural context [32]. In addition to comparing constructions of bulimia with anorexia, researchers have drawn attention to ways in which eating disordered behaviors are normative, rather than abnormal: binging and purging are not viewed as distinct from dieting and exercising for aesthetic or health reasons, with both made possible through the same social and historical conditions [41,42]. In a related area, social constructionist research has explored how constructions of anorexia work in conjunction with constructions of femininity, and in particular the idealised thin body that is so valued in women [43].

While research using social constructionist theory has been important in exploring some of the personal and social meanings of eating disorders, there are limitations. For instance, the focus is often on anorexia or eating disorders more generally, rather than bulimia and bulimic behaviors, specifically [40,42]. Further, much analysis is theoretical, and relies on secondary data such as media texts [39,40,42,44], or data from interviews with a relatively small sample [38,41]. Consequently, there is a need for further empirical work investigating social constructions of bulimia. This is the aim of the present study, which will use Q methodology to identify and describe the constructions of bulimia that circulate in a contemporary Western society, Australia. This will involve sampling a range of perspectives on the issue, held by individuals who have engaged in bulimic and other eating disordered behaviors, those who have had other personal experience of eating disorders (e.g. knowing a friend or family member to go through it), and those who have no personal experience with eating disorders.

### Q methodology

Q methodology combines both qualitative and quantitative techniques to study the “subjectivity involved in any situation” ([45], p. 561). As a method, it works to identify a range of subjective viewpoints that are shared by a number of individuals through an inverted form of factor analysis [46]. These participants’ *construction* of the issue can then be contrasted with that of other individuals, who share a different way of thinking about the topic [47]. In Q methodology the aim is to describe and interpret the point-of-view associated with each factor; as such, it is not “the ‘constructors’―the participants―who are the focus of the approach but the ‘constructions’ themselves” ([29], p. 180).

The strength of Q methodology is drawn from both qualitative and quantitative research traditions [48], and has been suggested as a more robust and suitable technique for exploring subjective viewpoints than alternative methods such as surveys [29,49–51]. It has been used to investigate the subjectivity involved in a variety of health issues, including understandings of irritable bowel syndrome [52], constructions of sex and intimacy in the context of cancer [53], parental judgement on infant immunisation [54], and the weight-control self-efficacy beliefs of obese women [48].

This paper presents the results from a Q methodological study, which explored constructions of bulimia. While the starting point for this research is that a number of alternative constructions of bulimia exist, the principle of “finite diversity” suggests that the potential range of such constructions is limited by socio-historical forces [29,51,55]. As such, it was expected that, through Q methodology, a number of coherent accounts of bulimia would emerge, and these would be shared among a group of participants.

## Methods

The first step in a Q methodological study is to produce a set of items or statements about the topic that participants will sort. This Q set is derived through exhaustive sampling, so as to be as representative as possible of the opinion domain [51]. Participants (the P set) are then chosen strategically, because of their ability to model a viewpoint on the topic through Q sorting, and because their viewpoint matters [29,51]. This study received ethical approval from the Human Research Ethics Committee of the University of Western Sydney, with protocol number H9909.

The participants rank items of the Q set according to their relative agreement or disagreement, such that each statement gains its full meaning only through how it is configured in relation to every other statement [56]. It is therefore the similarities across the overall arrangement of statements in participants’ Q sorts that are the basis for factors in the inverted factor analysis [51]. These processes are described below for the current study, with further description of the features and method of Q methodology provided elsewhere [51].

### Item sampling – the Q set

In creating the Q set, statements should be representative of the different perspectives circulating in the public domain about that topic [57]. A Q set should “cover all the ground within the relevant conceptual space” ([51], p. 58), while remaining balanced and unbiased to any particular viewpoint [51]. To this end, the Q set used in the present study was derived through sampling constructions of bulimia in a wide range of naturalistic contexts [58,59], including academic sources, media, websites and online communities. This sampling process, and further refinement of statements for the Q set, was completed by the first author, in consultation with the second and third authors. Sampling statements involved reading the first 20 academic papers on Google Scholar returned with the search term “bulimia”; these sources were supplemented by a larger literature review of bulimia carried out in preparation for this research. News media was reviewed through a web-based search of archived news reports (using Google News) and the websites of Australian print magazines that focus on health and/or women’s issues (e.g. Cleo). Additionally, any user comments sections associated with these articles were also reviewed. The top ten websites that were returned for a search of bulimia in Google were also reviewed to derive statements. These texts typically presented content on bulimia concerning health information and help-seeking. A web search was also used to identify visual depictions of bulimia and eating disorders, which lead to a pool of five American made-for-television films produced over the last 30 years on bulimia and a number of episodes of a popular Australian soap opera that aired in 2006. This content was all accessible online through the video sharing website YouTube, again allowing for a review of user comments and opinions on these videos. Finally, the social media website Tumblr, which allows users to aggregate content according to topics (tagging), was also reviewed for mentions of the word “bulimia”. This search resulted in an item-pool of approximately 200 statements about bulimia.

A structured approach was employed to reduce the number of items [51], conducted by the first author, through a process of discussion with the second and third authors. This involved organising items together based on their similar subject matter, and resulted in six fairly coherent themes, each comprising 20–30 statements. These themes were: bulimia as a problem, bulimia as a solution, bulimia as a choice, bulimia as a person, bulimia as normative, and bulimia as marginalised. Statements were then refined within each of these themes; this involved eliminating items that were repetitious, breaking up double-barrelled items, and editing and rewording statements to improve their readability [51]. In service of this, all statements used the term ‘bulimia’ rather than ‘bulimia nervosa’, as the latter is wordier, and arguably a more clinical and less widely known term.

The statement refining process resulted in a Q set of 54 items, which was piloted with volunteers using the web-based software Q-Assessor [60]. In response to their feedback on the items and the Q sorting task, this item pool was further shortened and refined to a final Q set of 42 statements.

### Recruitment and participants – the P set

In Q methodology, recruitment of participants is a strategic, rather than random, process [51]. Participants in this study were recruited through two channels to maximize the possibility that a variety of perspectives could be expressed [46]. The first group comprised 71 individuals (59 women, 9 men, *M*_age_ = 22, *SD* = 6, a further three did not provide demographic details), who identified as having at least a general knowledge of bulimia; they were recruited through undergraduate psychology courses. It is standard practice in Q methodology to include individuals in the P set whose viewpoints are of particular significance to the research focus [51]. The first group of this sample fulfills this brief as it is skewed towards younger women, the demographic in which eating disorders are most common [3,4]; further, as psychology students, these individuals are assumed to have some knowledge and interest in mental health. To further satisfy this recommendation, the second group of participants consisted of six women who reported engaging in bulimic behaviors (*M*_age_ = 20, *SD* = 3). They were recruited through advertising on social media and through print material in public sites on university campuses across western Sydney.

The final P set of 77 participants ranged in age from 17 to 50 (65 women, 9 men, *M*_age_ = 22, *SD* = 6). Three participants did not provide demographic details, something that may have been due to technical difficulties associated with online data collection, human error (e.g. missing a question or closing the window early), or to a resistance to providing any personal or potentially identifying information. While this sample size might be considered small for conventional factor analysis, the nature of the statistical technique in Q methodology, where the analysis is inverted, makes this sample perfectly adequate [46]. Indeed, the rule of thumb offered for use in Q methodology studies [29] indicates that a participant pool of between 40 and 60 is satisfactory for Q analysis. The majority of individuals in the P set indicated the cultural group with which they identified as Anglo-Australian (52%), followed by European (14%) and Middle-eastern (13%).

### Procedure – Q sorting

**Figure 1:**
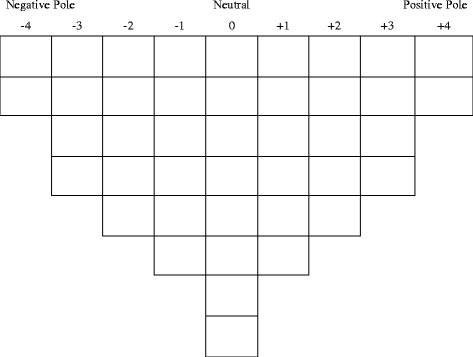
**Participant response grid (Q grid).**

Participants completed the Q sorting task online, using the website Q-Assessor [60]. On the introduction page they were asked to rank a set of 42 statements from those they most agreed (+4) with to those they most disagreed (−4) with, based on how well they represented their own understanding and thoughts about bulimia. The software directed participants through three stages of sorting. In the first, each participant organised the statements into three categories; those they agreed with, those they disagreed with, and those they were neutral about. At the second stage a fixed quasi-normal distribution (Figure 1) was introduced. This is a standard tool in Q sorting: the forced distribution simplifies later statistical procedures without impacting the factors that are produced [61], while the quasi-normal shape reflects that participants will feel comparatively strongly about a limited number of items [51]. Participants were first asked to sort the poles (+4 and −4); they were able to click through the statements, first in the “agree”, and then “disagree”, category, to select and “sort” the two they feel most strongly about. In the third stage, participants were free to rank the remainder of the statements into the distribution, moving them around and reviewing their placement up until they selected “submit”. Following the sorting task, participants were asked a series of open-response questions. These allowed participants to elaborate on how they found the task and why they sorted the statements in the way they did (particularly the poles). There were also a number of questions asking participants about their own understanding of bulimia, their level of personal involvement with the topic and how they came by most of their knowledge about bulimia.

### Q analysis and factor interpretation

Factor analysis is a common statistical method for classifying variables; in Q methodology these variables are participants’ Q sorts [61]. Through correlating in this by-person way “any sizeable portions of common or *shared* meaning” ([51], p. 98) across Q sorts are identified; these portions of shared meaning become factors.

Factor analysis was, conducted by the first and second author in collaboration, in Q-Assessor, which after correlating all Q sorts together, uses the centroid method to extract factors. This technique is favored by Q methodologists [51,61] and aims to account for as much of the study variance as possible with each successive factor that is extracted. Extracted factors were then subjected to a varimax rotation. This method positions factors so that the overall solution accounts for as much of the explained variance as possible, while having each Q sort load significantly on only one study factor [51]. Varimax rotation is accepted as a sound method of factor rotation and was a suitable choice in this study as it reveals “a subject matter from viewpoints that almost *everybody* might recognize and consider to be of importance” ([51], p. 126). Following this rotation, a factor was selected if it possessed an eigenvalue greater than one, and had at least two Q sorts loading significantly upon it [51,61]. This joint criteria is an accepted standard in Q methodology, reflecting the focus on *shared* meaning, where a shared viewpoint is one that is common to more than one individual [51]. Significant factor loadings were assessed using the Fuerntratt criterion, which is more stringent than many others used in Q methodology (see [51,61]), as it takes into account both the factor loading of a Q sort *and* its communality (i.e. the Q sort variance explained by all factors): a loading is significant if the amount of a Q sort’s variance explained by one factor exceeds 50% of the total variance explained (Fuerntratt, 1969, cited in [62]). Given that Q methodology is a novel approach exploring eating disorders, a more conservative criterion was considered appropriate. Six factors met these criteria, and were subjected to interpretation.

Interpretation of factors was facilitated by the creation of factor arrays; these are computed for each factor based on a composite of the Q sorts that load on it. By examining the meaning, ranking and overall configuration of statements in these arrays we begin to understand the construction of bulimia associated with that factor. The ranking of statements was compared across factors to determine similarities and points of divergence among the perspectives [51]. Finally, to supplement the interpretation, and more fully examine the context in which these constructions had come about, the socio-demographic data and the answers to the open-response questions about bulimia were examined for those participants who loaded significantly on a factor. This process of interpretation was initially conducted by the first author, with the second and third author making suggestions for elaboration and revisions, resulting in a final analysis which was agreed by all three authors.

## Results

The centroid technique extracted seven factors that accounted for 60.5% of the total variance in this study. After varimax rotation all seven factors had eigenvalues in excess of one (≥4.12), however, only six had at least two significant Q sorts. Construct validity for each factor was indicated by the composite reliability coefficient (r_c_), with all exceeding the acceptable value of 0.7. In Table 1 the characteristics for these factors are displayed. Table 2 shows the socio-demographic data for the participants whose Q sorts defined each factor. Factor scores of each statement across each factor are presented in the factor arrays in Table 3. In the following description of factors, bracketed notation is used to signify a statement’s ranking within a factor array; for instance “(5: −4)” indicates that statement 5 was ranked at the −4 (most disagree) position.

**Table 1:** **Q factor characteristics**

**Characteristic**	**Factor**					
	**A**	**B**	**C**	**D**	**E**	**F**
Number of defining sorts	4	7	4	7	2	2
Composite reliability	0.94	0.97	0.94	0.97	0.89	0.89
Eigenvalue	7.36	9.77	5.28	10.16	5.15	4.71
% of explained variance	9.56	12.69	6.86	13.20	6.68	6.11

**Table 2:** **Socio-demographic for Q sorts (participants) defining each factor**

**Variable**	**Factor A (** ***n*** =4)* (Count, %)	**Factor B (** ***n*** **=7) (Count, %)**	**Factor C (** ***n*** **=4) (Count, %)**	**Factor D (** ***n*** =7)* (Count, %)	**Factor E (** ***n*** **=2) (Count, %)**	**Factor F (** ***n*** **=2) (Count, %)**
Gender						
Woman	2 (100)	7 (100)	3 (75)	4 (66.7)	2 (100)	1 (50)
Man	0	0	1 (25)	2 (33.3)	0	0
Other	0	0	0	0	0	1 (50)
Age (*Mean, S.D.)*	19 (1)	22.3 (6.4)	20.3 (2.9)	19.8 (2.4)	18.5 (0.5)	20.5 (0.5)
Disordered eating experience						
Direct#	0	3 (42.8)	0	2 (33.3)	0	1 (50)
Friend/family member	1 (50%)	2 (28.6)	2 (50)	1 (16.7)	0	0
Impersonal	1 (50%)	2 (28.6)	2 (50)	3 (50)	2 (100)	1 (50)

*As some defining participants did not provide demographic data, calculations are based on the participants who did provide this information.

#Direct means that a participant stated they have engaged in bulimic behaviors.

**Table 3:** **Q-set statements and factor array**

**Item**		**Factor**					
		**A**	**B**	**C**	**D**	**E**	**F**
1	People with bulimia are distressed by their symptoms.	2	2	−1	1	0	0
2	Individuals with bulimia are victims of their disorder.	0	3	3	2	−1	1
3	Bulimia is a disease that gets worse over time.	4	0	−2	1	**3**	−1
4	People with bulimia don’t know why they binge and purge.	3	−1	−1	−1	0	0
5	For those with bulimia, food is a means of escape.	−3	2	3	−1	−1	2
6	A person with bulimia attempts to improve their mood by eating.	−3	0	**4**	0	0	0
7	Bulimia might start as a way for a person to express feelings they are afraid to deal with.	1	1	1	1	0	1
8	People with bulimia are afraid of living without their condition.	0	0	−2	0	−1	2
9	Binging and then purging might give a person with bulimia a sense of control.	−1	**3**	1	0	1	3
10	Many people with bulimia have experienced some sort of past trauma.	0	1	−1	1	−2	0
11	Bulimia is getting to eat what you want and still stay thin.	−2	−1	−2	−3	**3**	1
12	Bulimia is something someone might try out after hearing about it e.g. on TV, social media, friends.	2	−2	0	0	1	3
13	A person with bulimia could stop their behaviors if they really wanted to.	**−4**	**−4**	2	0	−1	−3
14	People with bulimia have failed to get anorexia.	−2	−3	−3	−2	−2	−1
15	Bulimia is a lifestyle choice.	**−3**	**−4**	**−3**	0	−2	1
16	People with bulimia want to recover.	3	0	0	1	1	−2
17	In bulimia, binging and purging are ways of punishing yourself.	1	**4**	−1	−1	2	−1
18	People with bulimia are success-orientated, seeking physical and mental control.	−2	1	1	−2	**4**	1
19	Dieting only develops into bulimia when a person has other predisposing characteristics.	0	−2	0	0	−1	**4**
20	Individuals with bulimia use food like addicts use drugs.	−1	1	2	−1	−2	−2
21	Binging is a sign of weakness in people with bulimia.	−2	−1	−1	0	0	−2
22	People with bulimia are obsessed with being thin.	1	0	1	2	1	0
23	People with bulimia are impulsive.	−1	1	−2	−1	−3	−1
24	People with bulimia are self-centered.	−3	**−3**	−1	−2	0	**−3**
25	Recovering from bulimia requires having self-control.	2	0	2	1	1	1
26	Purging after eating a big or fattening meal is no big deal.	−1	−3	0	**−4**	**−4**	−1
27	Bulimia is an understandable response in a society with lots of high calorie foods.	0	0	0	−2	−2	−3
28	Bulimia is an extreme example of the weight and eating concerns that affect many women in our society.	3	2	3	3	3	0
29	Bulimia is very different from dieting and normal concerns about shape and weight.	1	2	0	3	0	−2
30	Nobody really knows what normal eating, or a healthy relationship with food, is.	2	−1	−2	−2	**−3**	**3**
31	Society probably underestimates the number of men who have bulimia.	2	3	1	2	1	**4**
32	Bulimia occurs because our whole society has an attitude that supports eating disorders.	−1	−1	2	−3	**3**	0
33	Bulimia is more common in women because our culture puts more pressure on them to be thin.	1	1	**4**	**4**	**4**	2
34	Bulimia is something to be ashamed of.	−1	−2	**−4**	**−3**	0	**−4**
35	Bulimia is more socially acceptable than anorexia nervosa.	−4	−2	0	−1	−3	3
36	Bulimia is not that serious.	−2	**−3**	−4	**−4**	−3	**−4**
37	People with bulimia trade health for looking good.	1	−1	0	2	2	−1
38	Media portrayals of an ‘ideal’ body shape contribute to the occurrence of bulimia.	**3**	2	2	**4**	2	2
39	Our society’s increased focus on physical appearance will likely lead to a rise in bulimia in men.	0	3	1	2	−1	−2
40	Bulimia is not just a women’s problem.	**4**	0	3	3	2	0
41	Bulimia is biological in nature.	0	−2	−3	−3	**−4**	−3
42	Bulimia is a mental illness.	0	**4**	−3	3	2	2

Bolded scores are for statements with normalized factor scores **≥ ±**1.4, indicating the most powerful exemplars for each factor.

### Factor A: “bulimia as uncontrolled behavior”

Factor A explained 9.6% of the study variance. Four participants significantly loaded on this factor. Two were women aged 18 and 20, with one mentioning having a friend who had bulimia. Unfortunately, the two other factor exemplars did not supply any demographic data.

In Factor A, bulimia is positioned as a serious and distressing condition (1: +2; 36: −2; 3: +4) of being out of control of one’s behavior. Cultural standards of an ideal body shape are deemed to play a large role in bulimia’s development (38: +3; 33: +1), with people with bulimia seen as being obsessed with being thin (22: +1). Defining participants agreed that while bulimia affects both women and men (40: +4), and may be underestimated in men (31: +2), the desire for a thin body is ranked as being more typically an issue for women (28: +3). As one 18 years old defining participant’s response to a question about the gender disparity associated with eating disorders suggests, “men also care about the way they present themselves, but they do not seem to go to the extremes that women do”. For the perspective associated with Factor A, bulimia is about extremes: while issues with weight and shape are prevalent in our culture, the concerns and behaviors that characterise bulimia are considered to be far more extreme, and distinct from regular dieting and exercise (28: +3; 29: +1).

Participants defining Factor A endorsed the view that the path to bulimia might begin with individuals experimenting with binging and purging behaviors (12: +2), perhaps falsely believing that it will help them achieve their desired body shape, with the notion that this behavior works as a method to eat what you want and stay thin rejected (11: −2). Instead bulimia becomes increasingly severe (3: +4), and individuals lose their self-control (25: +2), becoming unable to stop their behavior (13: −4) and, ultimately, sacrifice their health (37: +1). In this vein, a defining participant (20 years of age) described a friend with bulimia as “they wanted to be thin and weren’t happy with how they looked, ended up in hospital over it”.

In the perspective associated Factor A, bulimia is characterised not only by a lack of control over one’s behavior, but also a lack of insight into it, with participants endorsing the view that people with this condition don’t understand why they binge and purge (4: +3). Bulimia is also positioned a distressing experience (1: +2), one from which recovery is desired (16: +3). However, as this requires a person to regain control over their behavior (25: +2), it is deemed to be difficult to achieve. This process, in which bulimia seems to begin with a choice but quickly becomes out-of-control, perhaps make the extent to which these individuals are victims difficult to judge (2: 0).

Participants defining Factor A prioritised sociocultural influences, while psychological issues were viewed as secondary (7: +1; 10: 0; 17: +1), or even rejected entirely, with food and eating not viewed as having any relationship to coping or mood improvement (5: −3; 6: −3). Thus bulimia is not about gaining control (9: −1), but about being unable to control one’s behavior, to the point of being unable to stop (13: −4). As this perspective considers bulimia in terms of behavior, and the lack of control over ones behavior, the possibility that its underlying nature is biological or psychological is either uncertain or unimportant (41: 0; 42:0).

### Factor B

Factor B accounted for 12.7% of the study variance. Seven participants had Q sorts that loaded significantly on this factor, with one being a negative relationship. These participants were all women, with an average age of 22.3 years and an age range of 18–37; three reported that they were engaged in bulimic behaviors currently, and another two had other personal experience (i.e. a close friend having bulimia). That one of the defining participants was negatively associated with this factor indicates that two coherent, but highly opposing perspectives on bulimia are subsumed under the one factor [51]. The first perspective to be considered is the one the majority of defining participants endorsed, the second is the converse position endorsed by only one of the participants who exemplified this factor.

### “Bulimia is a distressing mental illness”

The first perspective that emerged from Factor B was a highly psychological account of bulimia. Defining participants endorsed the view that bulimia is a serious mental illness (36: −3; 42: +4), distinct from normal practices of weight control and concerns about one’s body shape (29: +2; 30: −1). While sociocultural pressure for a thin body is deemed to have a hand in its development (38: +2; 28: +2), the desire for thinness is not positioned as the main issue in bulimia (22:0; 11: −1). Rather, participants defining this factor endorsed the idea that bulimic behaviors form as a means of dealing with upsetting issues in one’s life, such as past trauma (10: +1). This view is expressed in the open-response of one of the defining participants (18 years of age) “…for me at least, bulimic behavior isn’t solely due to concerns about my appearance – it’s a method of coping with overwhelming, negative feelings and self-loathing”. Further, as is suggested by this participant’s statement, individuals are considered to have some insight into their condition, and the reasons behind their bulimia, as defining participants disagreed with the idea that people in this state don’t know why they binge and purge (4: −1).

In this perspective, bulimia is positioned as a “coping mechanism”; defining participants endorsed the view that individuals may find solace in food (5: +2), and gain a sense of control from bulimic behaviors (9: +3). However, this situation is ultimately deemed to be maladaptive, as the experience of bulimia is positioned as distressing (1: +2), and bulimic behaviors are ranked as being self-punishing (17: +4). As bulimic behaviors both alleviate and contribute to distress, it is difficult to say whether or not people with bulimia want to recover (16: 0).

Participants defining the perspective of Factor B slightly endorsed the idea that the personality of the person dealing with bulimia has a role in their condition; they were, thus, likely to be high achievers (18: +1) or impulsive (23: +1). At the same time, and as with many biological illnesses, bulimia is positioned as something that anyone, man or woman, could develop (31: +3), as no predisposing characteristics are necessary (19: −2); an individual does not choose it (15: −4; 12: −2), has little control over their condition (2: +3), and is unable to just stop it (13: −4).

### “Bulimia is a way to lose weight”

In the second perspective to emerge from Factor B, bulimic behaviors are positioned as a method an individual, most likely a woman (31: −3), uses to lose weight (11: +1). While there may be particular characteristics that make someone more likely to engage in bulimia (19: +2), these are unlikely to be psychological in nature (42: −4; 17: −4; 9: −3). It is very much something that an individual has control over (13: +4); their choice to engage in binging and purging (15: +4; 12: +2) is motivated by a desire to improve their appearance (37: +1). As the open-response by the woman (19 years of age), who defined this perspective, indicates, “they like to look skinny to impress men”. This behavior is deemed to be quite shameful (34: +2), perhaps because it is viewed as selfish (24: +3), or because these individuals have failed at restricting their food intake, like an anorexic (14: +3), and binging is therefore a sign of weakness (21: +1). On the other hand, bulimia is not positioned as a serious problem (26: +3; 36: +3), and is more accepted socially than anorexia (35: +2).

### Factor C: “self-medicating with food”

Factor C explained 6.9% of total variance with 4 participants significantly associated with this factor, three of whom were women. The average age of these participants was lower than the overall sample at 20.3 years, with a range of 17–25. Two defining participants indicated that they knew a friend who engaged in bulimic behaviors.

The participants defining Factor C rejected the notion that bulimia is a mental illness (42: −3). Instead, individuals with bulimia are deemed to be the victims (2: +3) of a culture that basically encourages eating disordered behaviors (32: +2). Defining participants rankings suggest that this may be because of the way thin bodies are idealised (38: +2); therefore, while men experience bulimia (40: +3), from the viewpoint of participants defining Factor C, women are more commonly affected because they are usually under more pressure to be thin (33: +4; 28: +3). The ubiquity of a culture that provides the preconditions for an eating disorder means, for participants exemplifying this perspective, that all individuals are affected to varying extents; thus while they rejected the idea that there is no standard for a normal relationship with food (30: −2), they did not deem bulimia to be so far removed from commonplace practices of dieting and weight control (28: +3; 29: 0).

While similar sociocultural issues are emphasised in other factors, the perspective in Factor C is distinguished by the important role of food. Defining participants endorsed the view that people with bulimia use food to improve their mood (6: +4), to get a high (20: +2), and to withdraw from problems in their lives (5: +3). Thus food is positioned as having a medicinal quality; one defining participant (woman, 25 years of age), in her open-response to a question about how she acquired her knowledge of bulimia, described a friend who “every time she gets angry, she eats a huge amount of junk food, then she regrets it and vomits everything she ate”.

According to one of the defining participants (woman, 25 years of age) in Factor C, bulimia involves a “struggle between the desire to lose weight and binge eating”. This tension between longing for a particular body (22: +1) and *using* food makes compensatory behaviors necessary in this viewpoint. Thus purging behavior is considered secondary to the disordered eating and seemingly less problematic, as unlike the perspectives for every other factor, participants defining Factor C did not out rightly reject the idea that purging after a large meal was “no big deal” (26: 0). The cycle of binging and purging is something the person with bulimia is ultimately positioned as responsible for (13: +2) and so in order to recover they must regain control of their disordered eating (25: +2). On the other hand, while the idea that bulimia is a serious problem is endorsed in this perspective (36: −4), defining participants indicated that the behaviors are not necessarily distressing (1: −1) and suggested that the reasons behind them are not unknown to the person with bulimia (4: −1). As such, individuals with bulimia may or may not wish to recover (16: 0).

### Factor D: “the pathological pursuit of thinness”

Factor D accounted for 13.2% of the variance and was defined by seven participants. Four of these were women and two were men, with a further participant failing to provide socio-demographic data. The average age of these participants was again slightly lower than the overall average, at 20.5 years (range 18–25). Two of these participants, recruited as first year psychology students, indicated having engaged in some level of disordered, or what they considered to be distressing, eating behavior in the past, though they did not provide specific details. Another participant stated that they had a friend who had experienced both anorexia and bulimia.

In the perspective associated with Factor D, the way a thin body is prized in our culture is deemed to be the biggest influence on the development of bulimia (33: +4). In particular, the role of the media in creating this ideal, and then pressuring individuals to attain it, is heavily endorsed in both defining participants ranking of statements (38: +4) and their open-responses: “I think people have it because of the pressure from the media to look a certain way” (woman, 18 years of age), “they (Women) want to look ‘skinny’ because according to most of society (mainly the (entertainment) media), only skinny girls are attractive and more feminine” (man, 19 years of age). Thus for participants loading on Factor D, what separates people with bulimia from their normal and healthy peers is that they are overly influenced by these messages, to the point of obsession (22: +2). People with bulimia are also positioned as willing to go to more extreme and serious lengths to attain this physique (26: −4; 36: −4), trading their health for looking good (37: +2). Therefore, bulimia is conceptualised outside the realm of normal eating (30: −2), dieting and weight management practices (29: +3), and cannot be explained by prevalent socio-historical conditions (27:-2; 32: −3). This unequivocal stance can be contrasted with Factor C, where bulimic behaviors are not positioned as deviant or abnormal.

While the cause of these behaviors might be societal, for the participants defining Factor D, binging and purging constitute a mental illness (42: +3); as one of these participants (woman, 19 years of age) suggested, it “affect(s) not just the body, but the mind of the individual as well”. Thus bulimia is deemed to be a distressing experience (1: +1), with individuals becoming the victim of their own extreme desire for thinness (2: +2). This situation of being a victim of one’s desire is perhaps why, for the participants defining Factor D, the extent to which a person with bulimia has chosen to be in that state (12: 0; 15: 0), or has the ability to get out of it (13: 0), is difficult to judge.

In Factor D, defining participants position bulimia as a problem that affects both women and men (40: +3; 39: +2), and may even be underestimated in the latter group (31: +2). However, the ranking of statements also suggests that, as a thin body is much more a standard of beauty for women, bulimia is likely still more of an issue for them too (33: +4; 28: +3). Indeed, defining participants’ comments indicate that being a woman puts an individual at risk for bulimia, because women are more “self-conscious” and “sensitive to other people’s thoughts about their weight/shape” (man, 19 years of age), “paranoid about (their) appearance” (woman, 19 years of age), and tend “compare themselves to other women” (woman, 20 years of age). Therefore the pathological pursuit of thinness, which bulimia is positioned as in Factor D, is something more likely to be engaged in by women.

### Factor E: “being the best at being thin”

Factor E accounted for 6.68% of total variance and was defined by two participants; both were women, aged 18 and 19 years, and neither indicated having any firsthand experience with eating disorders.

This perspective is in some ways similar to Factor D, with bulimia again considered a mental illness involving the extreme pursuit of thinness (33: +4; 28: +3; 42: +2; 38: +2). However, there are subtle distinctions. For Factor D, those who develop bulimia are positioned as more *susceptible* to media and cultural messages; they are victims of these messages promoting a thin body, and the extreme behaviors they use to acquire it. On the other hand, the participants defining Factor E viewed people with bulimia not as victims (2: −1). Rather, these participants endorsed the idea that people with bulimia are seeking control (18: +4), particularly of their bodies because they are determined to be thin (9: +1; 22: +1). They are methodical in their attainment of this goal, with the idea that people with bulimia are impulsive rejected (23: −3). Bulimia is therefore positioned by the participants defining Factor E as a method that allows individuals to eat what they want while still achieving a thin body (11: +3). Though attempts to be thin become increasingly destructive (17: +2; 37: +2), the socially valued nature of this goal, and the measured way in which these individuals pursue it, perhaps makes it more difficult in this perspective than others to decide whether a person with bulimia is self-centred (24: 0).

For the participants defining Factor E, bulimia involves the rigid dedication to being thin, at any cost. While still conceptualised as a mental illness in this account, psychological issues are not viewed as important to either the development or maintenance of bulimia, with the notion that a person with bulimia experiences distress being treated neutrally or with mild disagreement (1: 0; 5: −1; 6: 0; 7:0; 8: −1; 10: −2), in contrast to Factor B. On the other hand, the rankings of defining participants suggest that as bulimia becomes worse as time goes on (3: +3), and results in the deterioration of health (37: +2), individuals with this condition generally want to recover (16: +1).

### Factor F: “extreme behavior vs. mentally ill”

Factor F explained 6.11% of the variance with two participants defining this factor; one of these participants was a woman of 20 years of age, while the other was a woman of 21 years who was recruited as part of the group that had engaged in bulimic behaviors. However, she did not label them as bulimia.

The perspective of the participants defining Factor F is in some ways similar to that of Factor A, with both positioning bulimia as something one chooses to begin (15: +1), due to pressures within culture (33: +2; 38: +2). However, while in Factor A bulimia is positioned as a behavioral problem, for those participants defining Factor F, it is conclusively positioned as a mental illness (42: +2). Indeed the ranking of a number of statements by the participants loading on Factor F suggests that it not so much bulimic behaviors that are the issue, but the underlying mental illness. For instance, defining participants suggested purging to be less problematic in this account than in most other factors, though it was still ranked negatively (26: −1). Further, the notion that bulimic behaviors are different from dieting and normal concerns about shape and weight is rejected (20: −2), with the whole notion of ‘normal’ being questioned (30: +3). Bulimic behaviors are also positioned as more socially acceptable than anorexia (35: +3). Thus, while bulimia forms around weight-management behaviors, for the participants defining Factor F, it may not be these behaviors that distinguish a person with bulimia, but other “predisposing characteristics” (19: +4). This idea is supported by one of the defining participants for Factor F, who reported engaging in binging, restricting, purging, and extensive amounts of exercise, but actively denied that they were bulimia or an eating disorder.

The factors distinguishing extreme eating behaviors from the mental illness bulimia are deemed to be psychological; defining participants endorsed the view that bulimia provides an individual with a means of coping with difficulties in their life (9: +3; 8: +2; 5: +2; 7: +1). As such, while for Factor F bulimia is likely underestimated in men (31: +4), an increased concern about physical appearance for men will not necessarily increase the number of men with bulimia (39: −2).

While bulimia is positioned as a mental illness by the participants defining Factor F, the notion that it is also an ongoing lifestyle choice (15: +1) receives more agreement here than in any other factor. This may be because people with bulimia are deemed to not really want to recover (16: −2), as bulimia provides not only a method of dealing psychologically with any issues, but also a means of staying thin while being able to eat what you want (11: +1). At the same time, even if a person with bulimia wanted to get better, it wouldn’t be so easy to just stop (13: −3), as the psychological issues involved in this condition make the thought of recovery frightening (8: +2).

### Consensus statements

Some of the statements were ranked in a similar way across factors. A number of items were included to examine individuals’ ideas about the gender disparity often found in statistics on the prevalence of eating disorders [63]. Two statements in particular (31 and 40) dealt with this issue. Though overall the ranking of each statement varied quite considerably across factors (31: +1 to +4; 40: 0 to +4), each factor ranked at least one of these statements at +2 or above, indicating an acknowledgement that men engage in bulimic behaviors. Across factors there was also a general agreement that bulimia is a serious issue (36: −2 to −4).

## Discussion

This research study explored cultural constructions of bulimia using Q methodology. As is anticipated within social constructionist theory, this investigation found that understandings of bulimia were not confined to one singular account, but made possible through a number of alternate constructions. Further, and in line with the Q methodological principle of finite diversity [29], the number of these constructions was limited, with six distinct but overlapping accounts of bulimia identified.

These constructions differ in a variety of ways, including influencing factors, how bulimic behaviors are positioned, and the extent to which the individual engaging in such behavior is viewed as responsible. By examining the configuration of statements within and across factors, in conjunction with the comments and demographic details of defining participants, a richer and more contextualized account for each factor was produced.

In the perspective of Factor A, entitled “bulimia as uncontrolled behavior”, individuals engaging in bulimia lack both control and insight into what they are doing. A potential implication of this construction is that they are unable to stop their behaviour alone, and, therefore, require the intervention of others to recover.

On the other hand, for Factor B, bulimia is not just about behaviour, but is the manifestation of an underlying mental illness. This conceptualization is common in both popular and specialized texts [64] and has been put forward by organizations that are authorities in the area of eating disorders [34]. It is also a position that is taken in Factors D, E, and F; however, Factor B is perhaps the most psychological account among these factors. This construction of bulimia as a dysfunctional coping mechanism is common in the research literature on eating disorders [65,66].

That almost half of the participants defining Factor B were women who reported engaging in bulimic behaviors suggests that these individuals may draw upon this construction to make sense of their own experiences. Perhaps, this is because this account offers a way of understanding bulimia that avoids blaming the individual, by positioning it as something separate from the self and outside normal behavior (a mental illness they carry within them), and gives high priority to emotional/psychological issues and the experience of distress. Thus, an appropriate response to individuals experiencing bulimia is compassion and help. Further, similar to the perspective of Factor A, the potential for a person with bulimia to exert agency, or recover alone from their condition, is foreclosed upon, as it is positioned as something outside of their control.

The other perspective associated with Factor B, defined by only one participant, has exactly the opposite implications. By positioning a person with bulimia as responsible for their condition, and able to recover if they wish, there is an explicit sense of blame not evident in other constructions, and also no implication that people with bulimia deserve sympathy.

For the understanding of bulimia in Factor C, entitled “self-medicating with food”, the mood improving qualities of eating are prioritized; this echoes an area of research that explores pleasure and the negative affect regulating capacities of food in bulimia [67,68]. This construction of bulimia positions the person as an agent of their disorder, but does so in a way that effectively minimizes blame (e.g. highlighting the functional aspects of binge eating as necessary mood improver, positioning the person with bulimia as a victim of their culture). As such, this construction might be useful, even emancipatory, for those persons engaged in bulimia, but it would appear that it is not readily drawn upon, as research finds considerable self-blame amongst these individuals [69].

The perspectives of Factors C and D both strongly endorse the influence of culture on bulimia, and particularly the idealization of a thin body; however, they do so to remarkably different effect. For Factor C, bulimic behaviors, in particular purging, are not positioned as especially abnormal or problematic. This can be contrasted with the perspective of Factor D, “the pathological pursuit of thinness”, where bulimic behaviors are viewed as unequivocally deviant. This clear-cut difference between normal and abnormal ties into how bulimia is commonly conceptualized in practice, where the categorical nature of diagnosis suggests that eating disorders are “discrete entities, demarcated by firm boundaries between one another and normality” ([31], p. S124). On the other hand this perspective conflicts with both feminist and social constructionist explanations of bulimia, and theoretical models in the research literature that view eating disordered behaviors as existing along a continuum with dieting and normal unrestrained eating (e.g. [70,71]).

The way in which bulimia is pathologized in Factor D may be less beneficial to people engaging in bulimic behaviors than other constructions identified here. Because it strongly connects bulimic behaviors with an extreme obsession with thinness, it positions the person as irrational, potentially even shallow, rather than, for instance, psychologically distressed, and thus worthy of sympathy or support (as in Factor B).

The construction of bulimia in Factor E, entitled “being the best at being thin”, there is a more explicit sense of blame, as the person with bulimia is positioned as actively and methodically engaging in behaviors that allow them to stay thin. The endorsement that a certain type of person develops bulimia is also present in research into personality traits and eating disorders [72]. The consistent nature of personality means that the potential for an individual within this construction to change ones behavior is inhibited with this construction.

Finally in the perspective of Factor F, “extreme behavior vs. mentally ill”, the issue of responsibility is determined by whether an individual is positioned as having the mental illness bulimia, or is simply engaging in behaviors that are culturally categorized as ‘bulimic’. There is a potentially dangerous implication of drawing upon this construction: if bulimic behaviours are not positioned as especially problematic, despite their associated health risk, then the likelihood of seeking help may be minimized.

While there are some points of convergence across a number of these perspectives, they remain fairly distinct. Only two issues were perceived similarly across factors: first that bulimic behaviors are also an issue for men, and second that bulimia is serious. The unanimous positioning of men within a construction of bulimia is interesting, and works against the idea that eating disorders are predominately, or only, an issue for women [5,73]. Thus any of the constructions used here may be readily drawn upon by men engaging in bulimic behaviors to make sense of their experiences. Also, endorsement of the idea that bulimia is serious amongst the defining participants for all factors is encouraging, given the health risks and psychological distress associated with such behaviors.

This research study has demonstrated that there are a multitude of different ways of understanding bulimia and bulimic behaviors. Social constructionist theory suggests that these different constructions are not inert, but have the potential to impact on the practices of those engaged in bulimic behaviors, and on those around them. That is, by providing a lens through which people understand behavior and experience, different constructions may open up or limit the possibility for certain kinds of actions.

The strength of this research lies in its combining of qualitative sensitivity to meaning with robust quantitative statistical analysis, to explore constructions of bulimia. However, it is only a first step. In no way is the sample of participants used in this study representative of the wider population. This is not a major concern for Q methodology, which aims to study constructions in their available diversity, rather than attempting to generalize about the proportions to which these constructions are distributed in the population [74]. However, to more fully explore the *range* of culturally circulating constructions of bulimia, it would be beneficial for future research to use a more diverse sample, in terms of age, gender and cultural background, as well as knowledge and experience of eating disorders. It would also be useful for future researchers to directly examine the relationship between constructions of bulimia and help-seeking behavior, combining Q methodology with other qualitative and quantitative research methods.

Constructions of bulimia will frame not only the experiences of those engaged in bulimic behaviors but also the experiences and behaviors of those around them. As such, it is important for future research to incorporate other stakeholders, such as family members and health professionals who deal with eating disorders. While it is entirely possible that these groups make use of similar constructions to those identified in this study, this is unknown. Indeed individuals sharing a similar socio-historical location in relation to this issue may draw upon one particular construction, as was the case here for women reporting bulimic behaviors defining Factor B; other studies have found similarly, with particular stakeholder groups becoming associated with one factor perspective (e.g. [53]).

## Conclusions

In conclusion, this study has shown that bulimia is not understood uniformly in our culture, but is a concept made sense of through a number of alternate constructions. The six constructions of bulimia that have been identified here share some things in common but have important differences too, such as in how much control/responsibility/blame the person is considered to have for their behavior or illness. These constructions potentially have distinct implications for how individuals engaged in bulimic behaviors think and behave, as well as how those around them may respond to them. As such, further study of constructions of bulimia may be able to provide insights into how and why, or why not, individuals seek help for their bulimic behaviors, and also the success of any treatment. While the assumptions of Q methodology hold that no one construction of bulimia is more valid than any other [33], some will certainly be more useful in reaching these goals.
